# The Identification of Large Rearrangements Involving Intron 2 of the *CDH1* Gene in *BRCA1/2* Negative and Breast Cancer Susceptibility

**DOI:** 10.3390/genes13122213

**Published:** 2022-11-25

**Authors:** Jihenne Ben Aissa-Haj, Hugo Pinheiro, François Cornelis, Molka Sebai, Didier Meseure, Adrien Briaux, Philippe Berteaux, Cedric Lefol, Gaëtan Des Guetz, Martine Trassard, Denise Stevens, François Vialard, Ivan Bieche, Catherine Noguès, Roseline Tang, Carla Oliveira, Dominique Stoppat-Lyonnet, Rosette Lidereau, Etienne Rouleau

**Affiliations:** 1 Department of Human and Experimental Pathology, Institut Pasteur de Tunis, Tunis 1002, Tunisia; 2 Laboratory of Biomedical Genomics and Oncogenetics, Institut Pasteur de Tunis, Tunis EL Manar University, Tunis 1002, Tunisia; 3 Faculty of Medicine, University of Porto, Rua Dr Roberto Frias s/n, 4200-465 Porto, Portugal; 4 University Hospital Center Gabriel-Montpied, Clermont-Ferrand, 58 Rue Montalembert, 63000 Clermont-Ferrand, France; 5 Department of Biology and Pathology, Laboratory of Cancer Genetics Institut Gustave Roussy, 94805 Villejuif, France; 6 Anatomopathological Service, Curie Institute, 26 Rue d’Ulm, 75005 Paris, France; 7 Oncogenetic Laboratory, Departement of Genetics, Curie Institute, 26 Rue d’Ulm, 75005 Paris, France; 8 Centre Leon Berard, 28 Promenade Léa et Napoléon Bullukian, 96008 Lyon, France; 9 Medical Oncology Department, Delafontaine Hospital, 93200 St. Denis, France; 10 Biostatistic Service, René Huguenin Hospital, Curie Institute, 35 rue Dailly, 92210 Saint Cloud, France; 11 Genetics Department, Intermunicipal Hospital Center Poissy St. Germain-en-Laye, 78300 Poissy, France; 12 Department of Cancer Anticipation and Monitoring, Clinical Oncogenetics, Paoli-Calmettes Institute, 13009 Marseille, France; 13 INSERM, IRD Laboratory, Economic and Social Sciences of Health & Processing of Medical Information, Aix Marseille University, 13009 Marseille, France

**Keywords:** breast carcinoma, gastric carcinoma, *CDH1* rearrangements, CNV, CGH array, *BRCA1/2* negative cases

## Abstract

E-cadherin, a *CDH1* gene product, is a calcium-dependent cell–cell adhesion molecule playing a critical role in the establishment of epithelial architecture, maintenance of cell polarity, and differentiation. Germline pathogenic variants in the *CDH1* gene are associated with hereditary diffuse gastric cancer (HDGC), and large rearrangements in the *CDH1* gene are now being reported as well. Because *CDH1* pathogenic variants could be associated with breast cancer (BC) susceptibility, *CDH1* rearrangements could also impact it. The aim of our study is to identify rearrangements in the *CDH1* gene in 148 BC cases with no *BRCA1* and *BRCA2* pathogenic variants. To do so, a zoom-in CGH array, covering the exonic, intronic, and flanking regions of the *CDH1* gene, was used to screen our cohort. Intron 2 of the *CDH1* gene was specifically targeted because it is largely reported to include several regulatory regions. As results, we detected one large rearrangement causing a premature stop in exon 3 of the *CDH1* gene in a proband with a bilateral lobular breast carcinoma and a gastric carcinoma (GC). Two large rearrangements in the intron 2, a deletion and a duplication, were also reported only with BC cases without any familial history of GC. No germline rearrangements in the *CDH1* coding region were detected in those families without GC and with a broad range of BC susceptibility. This study confirms the diversity of large rearrangements in the *CDH1* gene. The rearrangements identified in intron 2 highlight the putative role of this intron in *CDH1* regulation and alternative transcripts. Recurrent duplication copy number variations (CNV) are found in this region, and the deletion encompasses an alternative *CDH1* transcript. Screening for large rearrangements in the *CDH1* gene could be important for genetic testing of BC.

## 1. Introduction

Breast carcinoma (BC) remains the most common cancer among women worldwide. It is the first cancer worldwide with approximately two million new cases registered in 2020 (24.5%), with significant geographical distribution variations. It represents the first leading cause of mortality by cancer worldwide, causing 684,996 deaths in 2020 and accounting for 15.5% of all cancer deaths. In France, BC is the most common and the most deadly form of cancer among females; it represents the first most frequently diagnosed cancer with an incidence of 28% (58,083 new cases per year) and it is the first cause of death with a rate of 17.6% (14,183 deaths) in 2020 [1].

The etiology of most BC cases is unknown. However, there are many risk factors for this disease, such as gender, age, a family history of BC at a young age, late menopause, benign proliferative breast disease, and genetic pathogenic variants in genes such as *BRCA1/2* [2]. *BRCA1* and *BRCA2* are the most studied BC susceptibility genes that explain a significant proportion of both hereditary breast and ovarian cancers [3,4,5]. In addition to the aforementioned genes, more than 100 loci are known to be associated with BC risk [6]. In BRCA-negative families, a greater extent of BC is caused by high to intermediate penetrance of BC genes that explain the genetic predisposition to BC, including several syndrome-predisposing genes: *TP53* (OMIM 113721) (Li–Fraumeni syndrome; OMIM 151623), *PTEN* (OMIM 601728) (Cowden’s disease; OMIM 158350), *STK11* (OMIM 602216) (Peutz–Jeghers syndrome OMIM 175200), *NF1* (OMIM 613113) (neurofibromatosis; OMIM 162200), and *CDH1* (OMIM 192090) (hereditary diffuse gastric cancer syndrome “HDGC”; OMIM 137215) [2].

To date, according to the Human Gene Mutation Database (HGMD), more than 155 pathogenic variants resulting in the loss of function of the *CDH1* gene have been reported worldwide [7,8,9]. However, no hotspots have been characterized [10]. E-cadherin (OMIM: 192090), a *CDH1* gene product that belongs to the cadherin superfamily, is a calcium-dependent cell–cell adhesion molecule that plays a critical role in the establishment of epithelial architecture, maintenance of cell polarity, and differentiation [11]. Pathogenic variants in this gene are correlated with gastric, breast, colorectal, thyroid, and ovarian cancer. Loss of function of this gene is thought to contribute to cancer progression by increasing proliferation, invasion, and/or metastasis. The ectodomain of this protein mediates bacterial adhesion to mammalian cells and the cytoplasmic domain is required for internalization. This gene is present in a gene cluster with other members of the cadherin family on chromosome 16. The *CDH1* gene is a BC susceptibility gene with high penetrance. The implication of germline pathogenic variants in the *CDH1* gene has been discussed for BC, particularly lobular breast cancer (LBC). The relationship between BC and *CDH1* is very interesting because E-cadherin immunostaining is used to distinguish ductal and lobular breast lesions. Loss of E-cadherin expression in breast tumors has been associated with genetic and epigenetic alterations, including truncating pathogenic variants in the *CDH1* coding sequence, loss of heterozygosity, and methylation of the *CDH1* promoter. In the COSMIC database (http://www.sanger.ac.uk/genetics/CGP/cosmic/, Accessed on 23 September 2022), the percentage of pathogenic variants is 61% in lobular neoplasia, 15% in ductal in situ carcinoma, 31% in infiltrating lobular carcinoma (ILC) (67/215), and 5% in infiltrating ductal carcinoma (IDC) (11/216). However, not all variants reported in the COSMIC database resulted in inactivation of the *CDH1* gene. Recent studies provided evidence of LBC as the first manifestation of HDGC. Deleterious *CDH1* pathogenic variants have been identified in women with bilateral LBC without a family history of DGC. Novel deleterious *CDH1* alterations have also been identified, raising the question of whether LBC can be inherited as an independent E-cadherin syndrome [12,13,14].

Approximately 15–20% of BC is familial, meaning that affected women have one or more first- or second-degree relatives with the disease. The hereditary form in these families is considerable, especially in families with clustered occurrence of BC with low age at onset [14]. Pathogenic variants in the *BRCA1*, *BRCA2*, *CHEK2*, *TP53* and *PTEN* genes account for 5–10% of breast and ovarian cancer cases overall. The prevalence of *BRCA1* or *BRCA2* pathogenic variants varies considerably between ethnic groups and geographic areas. Population-specific pathogenic variants have been described in Iceland, the Netherlands, Sweden, Norway, Germany, France, Spain, countries in central and Eastern Europe, and Ashkenazi Jews [15].

The ongoing study “Understanding how *CDH1* germline pathogenic variants affect HLBC” [16] is a clinical genetic study aiming to identify the role of *CDH1* in HLBC without DGC aggregation. The initial aim of this study is to investigate the prevalence of *CDH1* pathogenic variants in women with early-onset (<45 or <50) invasive or in situ LBC, bilateral LBC, and LBC without a family history of HDGC. To date, 120 patients have been enrolled and six *CDH1* germline variants have been identified: one splice site variant with uncertain significance and five missense variants (three VUSs and two pathogenic). The identified VUS are currently being evaluated to assess their pathogenicity [17]. Other studies did not detect any deleterious *CDH1* pathogenic variants [18,19,20]. The recent large screening of families with LBC did not detect any deleterious variant in the *CDH1* gene [21]. However, some cases of families with multiple BC and isolated DGC have been reported. In one case, a case of synchronous ILC and DGC who had three family members with BC, mostly lobular, was reported [22]. Multiplex ligation-dependent probe amplification (MLPA) was used for detection and CGH-array for characterization [23]. Deletions were divided into five different classes: from the 5′ region to exon 2; from the 5′UTR to exon 1; exons 1 to 2 (in two families); exons 14 to 16; and from the last exon, 16, to beyond the 3′UTR. In two of these five classes, the breakpoints were located in the intron 2.

Because the association between *CDH1* pathogenic variants and BC has been questioned and E-cadherin expression has been associated with LBC, the present study examines the proportion of large *CDH1* rearrangements in probands with BC predisposition that are negative for pathogenic variants in the *BRCA1* and *BRCA2* genes. A dedicated CGH-array was used and the detected rearrangements were validated via transcript analysis.

## 2. Material and Methods

### 2.1. Patients and Data Collection

A cohort of 148 patients with BC, without any *BRCA1*/*BRCA2* pathogenic variants, was studied. All patients gave informed consent for molecular analysis of BC predisposition using an informed consent form. The analysis was limited to large rearrangements as putative events in BC predisposition.

The tumors were defined histologically as follows: 104 (70%) patients had IDC, 20 (14%) had ILC, 11 (7%) had in situ ductal carcinoma and 2 (1)% had an in situ lobular carcinoma. Eleven (7%) had other histologies (comedocarcinoma, tubular carcinoma, undifferentiated carcinoma). Nineteen percent of patients were younger than 40 years at initial diagnosis, and the rest were older than 60 years.

### 2.2. DNA and RNA Preparations

DNA was extracted from peripheral blood leukocytes using the Qiagen Maxi Kit (Qiagen SA, Courtaboeuf, France). Total RNA was extracted from lymphoblastoid cell lines via the acid phenol guanidine method using the RNA-BTM Q2 kit (Eurobio, Courtaboeuf, France). cDNA was generated using the random hexamer protocol from SuperScript™ III (Invitrogen, Carlsbad, CA, USA), and RT-PCR was performed as previously described [24].

### 2.3. Zoom-in CGH-Array

A zoom-in CGH array was designed specifically for the study of the *CDH1* gene and flanking sequences covered with 556 oligonucleotides in an Agilent^®^ Platform—home-designed array (Agilent Technology, Santa Clara, CA, USA). The MLPA kit SALSA P083-C1 *CDH1* (MRC Holland, The Netherlands) contains 17 probes for the *CDH1* gene, including two in exon 2. The flanking regions were covered in the 5′ region up to 99.46 kb and in the 3′ region up to 99.97 kb. In the zoom-in CGH-array, the average density of the probes ranged from 20 to 500 bp. All data were interpreted after standardization of the oligonucleotide log2ratio intensities in the *CDH1* locus, as previously described [25].

### 2.4. Breakpoint Sequencing

The CGH-array provided the coordinates of the breakpoint on the genomic sequence. Primers flanking the deletion and duplication were selected to amplify a specific region of the allele with the rearrangement. The PCR protocol, using Ampli-Taq Gold mix (Applied Biosystems, Courtaboeuf, France), was as follows: one denaturation cycle at 96 °C for 10 min, 40 cycles of 96 °C for 30 s, 60 °C for 30 s, 72 °C for 3 min, and 72 °C for 20 min. PCR products were analyzed on an agarose gel and then purified and sequenced in both directions using the PCR primers with the BigDye Terminator Cycle Sequencing Reaction Kit and an ABI Prism 3130XL automated sequencer (Applied Biosystems, Foster City, California 94404, USA). Sequence analysis was performed using the human reference sequences available in the UCSC genome browser (http://genome.ucsc.edu, accessed on 23 September 2022). Size and breakpoints were analyzed in comparison to CNV from the Toronto database of the Genomic Variants database (http://projects.tcag.ca/variation; accessed on 23 September 2022).

### 2.5. qPCR-HRM

Point mutations and large rearrangements in the *CDH1* gene were validated using a pre-screening quantitative PCR-high resolution melting (qPCR-HRM) approach on a LightCycler 480 (Roche Diagnostic, Pentzberg, Germany) as described previously [26]. Primers were selected to obtain an efficacy close to 2 with a dilution series for validation (upon request) and designed using Oligo6 software^®^ (Cascade, CO). Specificity was validated using a sequence compared to the reference sequence. Since *CDH1* germline mutations are rare, HRM screening for some amplicons was validated with somatic screening of in situ LBC from frozen tissue. qPCR-HRM was validated with DNA from in situ LBC tissue: c.385C>T, p.Gln129Stop (exon 3), c.406delC, p.Gln255ArgfsX27(exon 4), c.763delC, p.Gln136LysfsX79(exon 6), c.2262C>G, p.Tyr754Stop (exon 14), c.2261dupA, p.Tyr754X(exon 14), and previously reported polymorphisms in four exons: exons 1, 12, 13, and 16.

### 2.6. RNA Analysis

RNA was extracted from EBV-transformed lymphoblasts using the RNA-BTM Q2 kit (Eurobio, Courtaboeuf, France) according to the manufacturer’s protocol. cDNA was generated using the random hexamer protocol from SuperScript™ III (Invitrogen Carlsbad, ThermoFisher Scientific, California) as previously described [24]. The primers were set to explore the alternative transcript from the EST isolated in intron 2 to exon 3, 4, and 5 of the *CDH1* gene.

A dedicated RNASeq panel (Agilent, Les Ulis, France) was used to explore alternative splicing sites in intron 2 of the *CDH1* gene on the RNA extracted from EBV-transformed lymphoblast without any deleterious or probably deleterious *CDH1* variants.

### 2.7. Immunochemistry

Immunohistochemical detection of the E-Cadherin protein was performed on 4 µm tissue sections using a monoclonal E-cadherin antibody (clone EP700Y—Ventana Medical System, Tucson, AZ, USA). Staining was performed on the Ventana BenchMark XT automated slide stainer and visualized using the ultraView DAB Universal Detection Kit (Ventana). Slides were then counterstained with hematoxylin. Each immunochemistry was validated with a positive control sample consisting of a case of normal breast tissue and a negative control sample in which the primary antibody was replaced with non-human-reactive rabbit IgG.

### 2.8. Nomenclature

The *CDH1* reference sequence used was NM_004360.3. All rearrangements were described as NC_000016.8 based on their position in the build36/hg18 assembly.

### 2.9. Statical Analysis

All collected data in this study were descriptive without any statistical analysis.

## 3. Results

**Figure 1:**
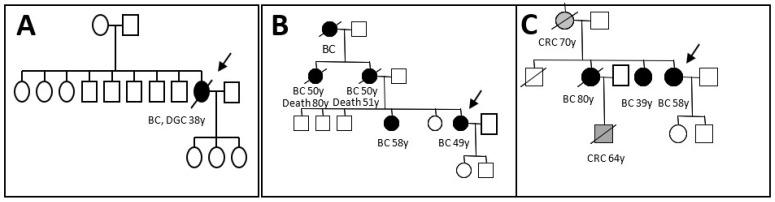
Family history of the three index cases harboring the exon 3, the intron 2 deletions and intron 2 duplication of the *CDH1* gene. (**A**) Pedigree of the index case’s family carrying the exon 3 deletion, with proband filled in black and indicated by arrow. (**B**) Pedigree of the index case’s family carrying the intron 2 duplication. (**C**) Pedigree of the index case’s family carrying the intron 2 deletion with ages of affected individuals at onset of disease in years (BC: breast cancer, CRC: colorectal cancer; black arrows: sampled individuals carrying identified variants).

A large rearrangement causing premature stop was detected in the exon 3 (chr16: 67,387,135–67,394,109) of the *CDH1* gene in a patient with bilateral LBC and DGC diagnosed at 38 years and deceased at the same age (Figure 1A). No family history of cancers was observed in this index case’s family.

**Figure 2:**
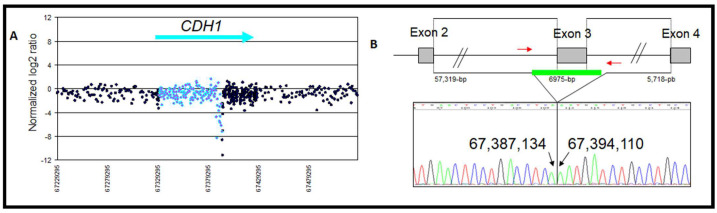
(**A**) Array CGH profile obtained for the heterozygous exon 3 deletion of the *CDH1* gene. Intron 2 is represented in blue dots. (**B**) Sequencing chromatogram of the mapped breakpoint for the exon 3 deletion from chr16: 67,387,135 to 67,394,109. Gray box: exons; green line: deletion locus. Red arrows: position of sequencing primers.

This exon 3 deletion was confirmed with qPCR-HRM sequencing and was located in locus chr16: 67,387,135–67,394,109, resulting in a 6975 pb deletion encompassing intron 2 c.164−5939 to intron 3 c.387+812 (Figure 2A,B).

No rearrangements were identified in the coding region of the *CDH1* gene in the other 147 screened samples. However, two large rearrangements (one duplication and one deletion) were detected in the intron 2 of *CDH1* gene in two different patients (Figure 1B,C).

**Figure 3:**
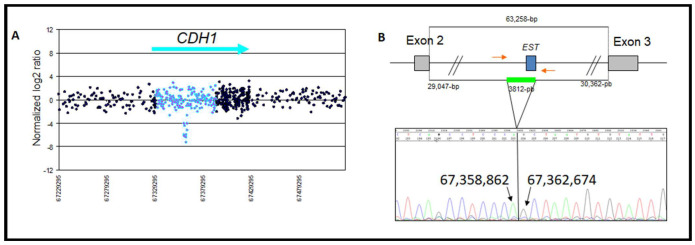
(**A**) Array CGH profiles obtained for the heterozygous intron 2 deletion of the *CDH1* gene. Intron 2 is represented in blue dots. (**B**) Sequencing chromatogram of the mapped breakpoint for the intron 2 deletion from chr16: 67,358,862–67,362,674 (NM_0043603: c.163+29048_c.164−30401del). Gray box: exons; blue box; EST; green line: deletion locus. Red arrows: position of sequencing primers.

A deletion of 3812 bp including part of intron 2 was located at chr16: 67,358,863–67,362,674 from c.163+29048 to 164−30362 (Figure 3A,B). The patient had ductal invasive BC at the age of 58. She had two sisters diagnosed with BC, one of them diagnosed at the age of 39. The other one died at the age of 80, with a daughter who developed a CRC at the age of 64. Her mother was diagnosed with CRC at the age of 70 (Figure 1C). This intron 2 rearrangement deleted the expressed sequence tag BP232006, which was found to be an alternative splice exon in a short form of *CDH1* containing exon 3 and a part of the exon 4. The alternative exon 1 (EST BP232006) and exon 3 were screened for point mutations in the 147 probands by qPCR-HRM, and none were found.

**Figure 4:**
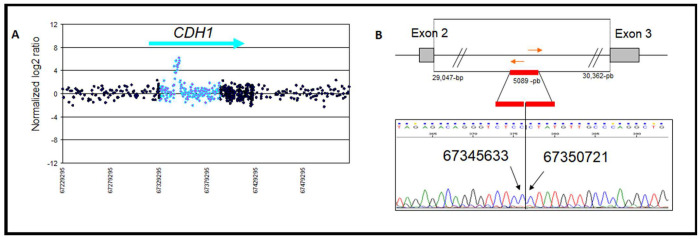
(**A**) Array CGH profiles obtained for the heterozygous intron 2 duplication of the *CDH1* gene. Intron 2 is represented in blue dots. (**B**) Sequencing chromatogram of the mapped breakpoint for the intron 2 duplication from chr16: 67,345,633–67,350,721(NM_0043603: c.163+15817_c.163+20905dup). Gray box: exons; red line: duplication locus; red arrows: position of sequencing primers.

**Figure 5:**
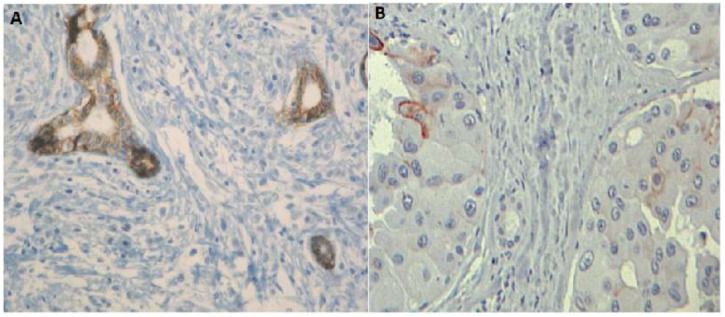
(**A**) Immunohistochemical staining for E-cadherin in bilateral invasive lobular carcinoma, showing an homogenous loss of E-cadherin expression and presence of E-cadherin in normal tissue X 200. (**B**) Immunohistochemical staining for E-cadherin in in situ ductal and lobular carcinoma, showing a heterogeneous loss of E-cadherin expression X 200.

A duplication of 5089 bp, including part of intron 2, was located on chr16: 67,345,633–67,350,721 from c.163+15817 to 163+20905 (Figure 4A,B). No EST was identified in this region; however, it is rich with transcription factor sites such as IRF4, NFKB, EBF, SP1, etc. (http://genome.ucsc.edu/ENCODE/; Accessed on 23 September 2022) (Figure S1). The BC histology of the patient carrying this duplication was a mixture of lobular and ductal in situ carcinomas; the patient was diagnosed at the age of 49 and died after one year. Her sister, her mother, and her aunt were diagnosed with BC at the age of 50 (Figure 1B). This index case demonstrated heterogeneous loss of E-cadherin expression in breast tumor tissue (Figure 5B).

In the RNASeq results, in this region, three alternative splice sites were identified in the start of intron 2: c.163+18993, c.163+19303, and c.163+19994 (Table S1) [27].No alternative start was identified near the expressed sequence tag BP232006.

The bilateral LBC with the germline exon 3 deletion had unambiguous loss of E-cadherin expression in the tumor cells (Figure 5A). The invasive ductal BCs with the germline rearrangement in intron 2 demonstrated similar heterogeneous staining, with some cells having strong and complete expression of E-cadherin and others have low and incomplete staining, while the positive control sample demonstrated uniform and intense membrane expression (Figure 5B). This staining pattern was performed twice in multiple slides and confirmed by two pathologists.

## 4. Discussion

The contribution of CNVs in BC risk remains undefined compared with the documented association between SNPs and BC susceptibility. In this screening of 148 patients predisposed to BC, with no deleterious variants in *BRCA1/2*, we detected a deleterious large germline rearrangement in the *CDH1* gene: c.164−5939_387+812del (Figure 2A,B), leading to the loss of the exon 3 and the production of a truncated protein (p.Val55GlyfsX38) as well as two large rearrangements in intron 2 (deletion and duplication) (Figure 3 and Figure 4). The classification of the identified CNVs was performed according to the recommendation of American College of Medical Genetics and Genomics (ACMG) and Clinical Genome Resource (ClinGen) [28]. The *CDH1* rearrangements and copy number variants (CNVs) reported in this study are listed in Table 1, along with the 15 previously reported CNVs (five duplications and 10 deletions) (Table 2). Eight variants affecting coding exons could be considered deleterious, although some were reported as CNVs in the Toronto database without available clinical data and four CNVs were reported in intron 2. The deletion of exon 3 was previously reported in a Japanese study including 13 patients with familial gastric carcinoma (FGC) using a combination of MLPA and RT-PCR analyses [29]. This mutation was identified in a 25-year-old woman diagnosed with FGC and having a GC familial history. Her brother developed GC and died at the age of 22. The previously mentioned study described the variant p.Val55GlyfsX38 as a variant involved in carcinogenesis in Japanese patients with FGC.

However, this is the first study reporting exon 3 deletion in the *CDH1* gene in patients with BC. Two possible mechanisms could be considered. Non-allelic homologous recombination (NAHR) may have recombined an AluSx sequence in intron 2 (5′ region) with an AluSx in intron 3 (3′ region). Non-homologous end-joining could have formed the deletion across an 11 nucleotide microhomology present in the 5′ and 3′ regions. The 5′ breakpoint was 279 bp away from a breakpoint associated with an exon 1–2 deletion, which could be a recombination hotspot due to two Alu sequences being close to each other [23]. The patient with the exon 1–2 (Table 2) deletion had a bilateral LBC with metastasis at the age of 32 years and died of DGC 3 years later. She came from a large family with eight siblings, but none had cancer. E-cadherin expression was not detected in the bilateral LBC using IHC.

Two of the three rearrangements reported in our study are located in intron 2 (Table 1 and Table 2), the largest intronic region in the *CDH1* gene. There are many conserved sequences in this intron involved in the initiation and maintenance of *CDH1* gene transcription [30]. A variant located in the intron 2, c.163+37235G>A (g.67,367,050), was associated with GC risk using an OR 4.55 (IC 2.09–9.93) [31].

For the deletion located in intron 2, EST BP232006 has been described in fetal muscle [32]. Exon 3 is conserved and there is an alternative *CDH1* transcript consisting of exons 1 (EST), 2, and 4. The alternative transcript appears to be untranslated as it does not have a unique open reading frame (codon stop in the three reading frames starting from the first intron). In the physiological state, this non-coding RNA could contribute to the transcriptional or translational regulation of the *CDH1* gene. In the pathological state, with deletion of the first exon and its promoter region as reported, the absence of this noncoding transcript could deregulate the expression and the function of the *CDH1* gene. No additional point mutation was detected in EST BP232006 in the 148 patients studied here. No EST was reported for the identified duplication in intron 2. A 21-nucleotide microhomology was present in the 5′ and 3′ regions and could explain non-homologous recombination. Two other duplication CNVs have been described in this region (CNV 77387 and 67021; Table 2). At the breakpoint of these three duplications, there are four homologous regions with at least 79% homology ranging in a size from 283 to 318 bp that could favor recombination in this region. Multiple transcription sites have been described in this intron (http://genome.ucsc.edu, Accessed on 23 September 2022). Our results highlight that this hotspot of recombination should be further investigated as four CNVs (three duplications and one deletion) are located near chr16: 67,347,663–67,348,065. Surprisingly, only one duplication (0.6%) was identified, and the duplication ranged from frequency from 5 to 10% (Table 2). Ultimately, this region may play a role in the regulation of *CDH1* gene expression.

No single nucleotide polymorphism (SNP) was found in the intron 2 deletion and duplication to evaluate the allelic disequilibrium and investigate the effects on transcription of the *CDH1* allele. Estimation of allelic imbalance in the *CDH1* transcription provided insight into the impact of these events [33]. RNA analysis for these intron 2 rearrangements revealed at least three variants in splicing sites between exons 2 and 3 [27]. Three variants were within the intron 2 duplication (Table S1).

The family history of the two patients harboring the intron 2 rearrangements (Figure 1B,C) reported multiple BCs in the families with an age of onset of more than 45 years, but neither GC nor LBC was diagnosed. For the intron 2 deletion (Figure 1C), the patient had ductal in situ carcinoma at 49 years and ductal invasive carcinoma at 53 years. For the intron 2 duplication, the patient had a mixture of lobular and ductal in situ carcinomas with few ductal invasive carcinomas at age 58 (Table 1). The E-cadherin IHC was performed on BC tissues harboring the intron 2 rearrangements and revealed heterogeneous staining (Figure 5B). These results were unusual in ductal carcinomas. Total inactivation of the E-cadherin could not be confirmed by loss of heterozygosity (LOH) analysis because no frozen tissue was available for this study. Finally, since IHC appears to be borderline for both intron 2 rearrangements, no argument could help to classify intron 2 duplication and deletion, which continue to be considered CNVs.

A recent study investigated NGS-based multiple gene panel sequencing in combination with a high-resolution CGH-array with the aim of identifying genetic risk factors for hereditary BC in 148 high-risk patients previously tested negative for pathogenic *BRCA1/2* variants. A large deletion in intron 2 was identified in one patient using MLPA analysis [34]. Indeed, exon 2 is involved in the propeptide part of the E-cadherin; alterations in this region may lead to impaired production and dysfunction of this protein. Oliveira’s study found several large deletions in exons 1, 2, 14, 15, and 16 (Table 2) of the *CDH1* gene in HDGC families [23]. According to previous studies, about 5% of HDGC probands have large deletions in the *CDH1* gene [23,29].

This is the first large CGH-array screening for large *CDH1* rearrangements in families with BC predisposition. The MLPA kit (SALSA P085 *CDH1* MLPA kit, MRC Holland) covers only the exonic regions of the *CDH1* gene, which did not reveal the intron rearrangements detected with CGH array. Further functional assays and cosegregation analysis should be performed to confirm the effect of all identified variants. In addition, our study has some different caveats and limitations, as the patient recruitment was not specifically enriched in LBC and could limit the yield of events. Access to the familial history and the possibility to propose co-segregation was limited, as no deleterious variant was detected to assure the family’s follow-up. Finally, assays to detect and characterize large rearrangements in the intron 2 of the *CDH1* gene are evolving and molecular exploration will be needed to understand the etiology of the other 145 BC cases with no *BRCA1/2* and *CDH1* pathogenic variants with the third generation sequencing NGS [35] and the target-enriched Nanopore sequencing [27,36].

**Table 2:** Summary of the *CDH1* rearrangements and CNVs reported in this study and in the literature. In bold, the breakpoint in intron 2 of *CDH1* gene and underlined are common regions found in at least two rearrangements. In the start and end column are reported the nature of the boundary and the presence of repeated motifs.

N	Rearrangements	Rearrangement Coordinates (hg18/GRCh36)	Gain/Loss	Reported Frequency	Size (bp)	Start#	End#	Classification	Reference
1	Exon 3 deletion	chr16: **67,387,135**–67,394,109	Loss	1/148	6975	Intron 2 AluSx	Intron 3 AluSx	Deleterious c.164-5939_387+812del, p.Val55GlyfsX38	This study and [29]
2	Full *CDH3* sequence and *CDH1* exon 1–2 deletion	chr16: 67,193,822–**67,387,415**	Loss	2/93	193,594	5′ region AluSp	Intron 2 AluSg	Deleterious c.-124-u134874_164-5659del, p.	[23]
3	Exon 1–2 deletion	chr16: 67,324,886–**67,330,557**	Loss	1/93	5672	5′ region AluSx	Intron 2 AluSg	Deleterious c.-124-u3810_163+742del, p.	[23,37]
4	Intron 2 deletion	chr16: **67,358,862**–**67,362,674**	Loss	1/148	3811	Intron 2 AluSx	Intron 2 AluJo	CNV	This study
5	Intron 2 duplication	chr16: **67,345,633**–**67,350,721**	Gain	1/148	5089	Intron 2 AluJo	Intron 2 FLAM_C	CNV	This study
6	Intron 2 CNV 67021	chr16: **67,345,074**–**67,351,437**	Gain	24/450	6364	Intron 2	Intron 2	CNV	[38]
7	Intron 2 CNV 77387	chr16: **67,345,117**–**67,348,065**	Gain	9/90	2949	Intron 2 MER-53	Intron 2	CNV	[39]
8	Intron 2 CNV 88182	chr16: **67,347,663**–**67,348,065**	Loss	1/90	403	Intron 2	Intron 2	CNV	[39]
9	Intron 2 CNV 5831	chr16: **67,330,369**–**67,331,573**	Loss	1/36	1205	Intron 2 AluJo	Intron 2	CNV	[40]
10	Exon 1 deletion	chr16: 67,328,695–67,328,844	Loss	1/93	150	AluJo	Intron 1 AluJo	Deleterious	[23]
11	Exon 14–16 deletion	chr16: 67,416,845–67,424,923	Loss	1/93	8078	FLAM_C Intron 13	3′ region AluJb	Deleterious	[23]
12	Exon 16 deletion	chr16: 67,424,298–67,425,126	Loss	1/93	828	AluSq Intron 15	3′ region AluJb	Deleterious	[23,37]
13	Exon 4–16 duplication CNV 77388	chr16: 67,397,988–67,426,849	Gain	1/90	28,862	Intron 3 MIR	3′ region	Deleterious c.388-1840_*1946del, p.Ala130MetfsX155	[39]
14	Exon 13–14 duplication CNV 9761	chr16: 67,414,790–67,420,815	Gain	1/112	6026	Intron 13	Intron 14	Deleterious c.1937-13_2296-243del p.Gln647ValfsX10	[41]
15	Intron 15 CNV 67022	chr16: 67,421,302–67,424,310	Loss	5/450	3009	Intron 15	Intron 15	CNV	[38]

## 5. Conclusions

To the best of our knowledge, this is the first CGH-array screening of the entire genomic sequence of the *CDH1* gene in BC predisposition reporting rearrangements in exon 3 and intron 2 of the *CDH1* gene. This study confirms that large *CDH1* rearrangements should be investigated in cases with the combination of BC and DGC. However, the role of the *CDH1* gene in families with only BC remains unclear. Other types of events could be investigated, especially in intron 2.

In conclusion, our study reveals novel variants in the *CDH1* gene in European patients with BC. Our data support the hypothesis that *CDH1* variants, and particularly the variants described, should also be considered in sporadic cases of GC and familial/sporadic BC. The presence of these variants in patients raises important questions regarding genetic counseling and diagnostic testing in DGC and BC patients.

## Figures and Tables

**Table 1:** Summary of the *CDH1* rearrangements and copy number variants reported in this study.

Gene		*CDH1*		
Exon/Intron		Exon n°3	Intron n°2	
Zoom in gene region (hg18/GRCh36)		chr16: 67,387,135–67,394,109	chr16: 67,358,862–67,362,674	chr16: 67,345,633–67,350,721
Type of rearrangement		Heterozygous deletion of 6975 pb	Heterozygous deletion of 3812 bp	Heterozygous duplication of 5089 bp
c. position		c.164-5939_387+812del	c.163+29048_164-30362del	c.163+15818_163+20906dup
Clinicopathogical characteristicsof the patient	Age at diagnosis	32	58	49
	Personal history	Bilateral lobular BC and metachronous diffuse gastric carcinoma.	Ductal Invasive BC	A mix of lobular and ductal In situ carcinomas.
	Familial history	No history	BC/CRC	BC
E-Cadherin expression		Homogeneous loss (Figure 5A)	Heterogeneous loss	Heterogeneous loss (Figure 5B)
Protein change		p.Val55Glyfs*38	-	-
Classification		Deleterious	CNV	CNV

## Supplementary Materials

The following supporting information can be downloaded at: https://www.mdpi.com/article/10.3390/genes13122213/s1, Table S1: Detailed information of the identified alternative splice sites. Figure S1: Transcription factor binding sites located from 67,345,633 to 67,350,721 of the identified duplication.
