# Supplementary figures and images for: The Identification of Large Rearrangements Involving Intron 2 of the CDH1 Gene in BRCA1/2 Negative and Breast Cancer Susceptibility
Source: Genes (Basel). 2022 Nov 25;13(12):2213. doi: 10.3390/genes13122213 (PMC9778491; doi:10.3390/genes13122213)

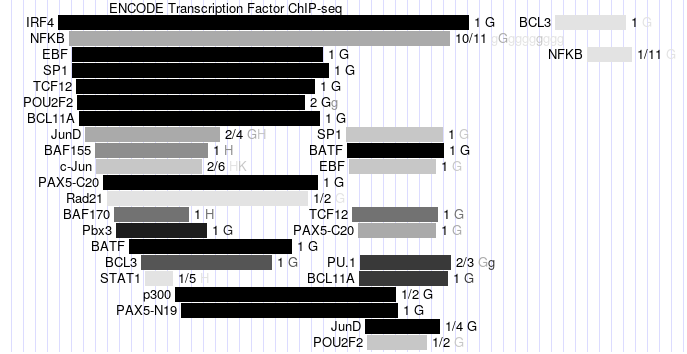

Supplement: Supplementary file 1 [file genes-13-02213-s001.zip › Figure S1.png]
